# A Minimal Intervention to Promote Smoke-Free Homes among 2-1-1 Callers: North Carolina Randomized Effectiveness Trial

**DOI:** 10.1371/journal.pone.0165086

**Published:** 2016-11-02

**Authors:** Rebecca S. Williams, Jana H. Stollings, Łucja Bundy, Regine Haardörfer, Matthew W. Kreuter, Patricia Dolan Mullen, Mel Hovell, Marti Morris, Michelle C. Kegler

**Affiliations:** 1 Center for Health Promotion and Disease Prevention, The University of North Carolina at Chapel Hill, United States of America; 2 Lineberger Comprehensive Cancer Center, The University of North Carolina at Chapel Hill, United States of America; 3 Department of Behavioral Sciences and Health Education, Emory Prevention Research Center, Rollins School of Public Health, Emory University, Atlanta, Georgia, United States of America; 4 Health Communication Research Laboratory, Washington University, St. Louis, Missouri, United States of America; 5 Center for Health Promotion and Prevention Research, University of Texas School of Public Health, Houston, Texas, United States of America; 6 Center for Behavioral Epidemiology and Community Health, Graduate School of Public Health, San Diego State University, California, United States of America; 7 United Way of North Carolina, NC 2-1-1, Cary, North Carolina, United States of America; Centre for Addiction and Mental Health, CANADA

## Abstract

This study examined the extent to which delivery of the minimal Smoke-Free Homes intervention by trained 2-1-1 information and referral specialists had an effect on the adoption of home smoking bans in low-income households. A randomized controlled trial was conducted among 2-1-1 callers (n = 500) assigned to control or intervention conditions. 2-1-1 information and referral specialists collected baseline data and delivered the intervention consisting of 3 mailings and 1 coaching call; university-based data collectors conducted follow-up interviews at 3 and 6 months post-baseline. Data were collected from June 2013 through July 2014. Participants were mostly female (87.2%), African American (61.4%), and smokers (76.6%). Participants assigned to the intervention condition were more likely than controls to report a full ban on smoking in the home at both 3- (38.1% vs 19.3%, *p* = < .001) and 6-month follow-up (43.2% vs 33.2%, *p* = .02). The longitudinal intent-to-treat analysis showed a significant intervention effect over time (OR = 1.31, *p* = .001), i.e. OR = 1.72 at 6 months. This study replicates prior findings showing the effectiveness of the minimal intervention to promote smoke-free homes in low-income households, and extends those findings by demonstrating they can be achieved when 2-1-1 information and referral specialists deliver the intervention. Findings offer support for this intervention as a generalizable and scalable model for reducing secondhand smoke exposure in homes.

## Introduction

Developing a minimal intervention that effectively promotes smoke-free homes in a range of populations and tobacco control contexts is important, as homes are a main source of secondhand smoke (SHS) exposure.[1] SHS causes lung cancer, coronary heart disease, and stroke in nonsmoking adults and, in infants and children, causes asthma attacks, respiratory and ear infections, and sudden infant death syndrome.[2, 3] Annually, SHS exposure leads to an estimated 600,000 deaths worldwide and, in the US alone, over 41,000 deaths among nonsmoking adults, 400 infant deaths, and an estimated $5.6 billion loss in productivity.[2, 4]

The current trial is the second in a series of studies aimed to develop, test, replicate and disseminate a minimal intervention to create smoke-free homes in community settings. Our recent vanguard RCT of the Smoke-Free Homes (SFH): Some Things Are Better Outside Program tested a minimal intervention among callers to United Way 2-1-1 of Greater Atlanta, a social services information and referral helpline.[5] This efficacy trial was built on formative research on family dynamics related to establishing home smoking bans,[6, 7] a pilot study,[8] and a multi-state survey of 2-1-1 callers showing a relatively low prevalence of smoke-free home bans.[9] The objective of the current North Carolina effectiveness trial was to examine the extent to which delivery of the minimal Smoke-Free Homes intervention by 2-1-1 information and referral (I&R) specialists had an effect on the adoption of home smoking bans.

In the vanguard RCT, 2-1-1 staff conducted study recruitment and enrollment, while intervention and follow-up interviews were conducted by university personnel.[5] Findings showed a 15 percentage point difference favoring the treatment over control group in households adopting home smoking bans at 3 and 6 months post-baseline (with bans validated via air nicotine monitors at 3 months).[5] The reduction in SHS exposure was similar to effects demonstrated in previous more intensive smoke-free home policy intervention studies.[5, 10–15]The Atlanta trial set the stage for tests of generalizability of the intervention in more typical 2-1-1 settings, wherein the intervention is provided by trained 2-1-1 staff.

Considerable attention has been paid in recent years to the gap between research and practice in public health.[16–19] A potential strategy for narrowing this gap is to focus more attention on the external validity of interventions.[17, 18, 20] Although an intervention tested in an efficacy trial with tight controls and intervention delivery by university staff may show an impact, the extent to which that impact will generalize across varied settings, contexts and populations should be established.[18, 19] Important steps in this process include testing interventions in real-world settings with non-research staff delivering the program, identifying core elements of an intervention, confirming its theory of change, assessing cost-effectiveness, and testing generalizability across different populations.[21–24] For SHS exposure in the home, the latter means different geographic regions with varied tobacco control contexts, diverse racial/ethnic groups and varied household compositions.

2-1-1, the delivery setting for this Smoke-Free Homes intervention, is a national information and referral service connecting individuals to community social and health services, reaching approximately 93% of the US population, and addressing 15 million calls per year.[25] Relative to the general population, 2-1-1 callers are disproportionately low-income, unemployed, uninsured, have fewer years of education, higher smoking rates, and lower likelihood of having a home smoking ban.[9, 26, 27] Providing extensive reach to vulnerable populations, and staffed by professionally trained I&R specialists, 2-1-1 organizations are strategic partners for testing, delivering, and ultimately sustaining interventions to reduce risk and improve the lives of low-income persons.[26] Thus, 2-1-1 call centers provide an excellent setting for assessing the efficacy, effectiveness, and generalizability of health interventions for vulnerable low income populations with higher risk of exposure to smoking and secondhand smoke.[17, 18]

Although SHS exposure in US nonsmokers was reduced by half from 1999–2000 to 2011–2012, 58 million people were still exposed.[28] The highest exposure and the fewest rules restricting smoking in the home existed among households with young children, non-Hispanic blacks, persons living below the poverty line, with less education, and/or living in rental housing, a demographic profile which correlates with the client population of 2-1-1.[1, 28–32]

Epidemiologic research has shown that nonsmokers and children living in homes that allow indoor smoking have disproportionately higher SHS exposure compared to those living in homes that do not allow smoking inside.[1, 33–35] Home smoking bans benefit both smoking and non-smoking residents, with lower levels of SHS exposure, fewer cigarettes smoked, and more attempts to quit smoking. [34, 36–41] Researchers have tested the effects of counseling parents of children with asthma, infants, or medically compromised children on exposure levels,[11–15, 42–44] and typically such interventions have taken place in or involved participant recruitment through clinical settings.[10, 14, 15, 43]

The purpose of the current study was to examine external validity of the community-based Smoke-Free Homes: Some Things are Better Outside intervention by assessing whether delivery by 2-1-1 I&R specialists, rather than research staff, is effective in supporting households to adopt home smoking bans.

## Methods

Participants were callers to United Way of North Carolina 2-1-1’s central call center between June and December 2013. It is the larger of two 2-1-1 NC call centers, covering 63 of 100 NC counties and receiving an average of 341 calls per day during the enrollment period. This study was a randomized controlled trial with assessments at baseline, 3 months, and 6 months post-baseline conducted from June 2013 through July 2014.

Four FTE 2-1-1 I&R specialists underwent one day of specialized training to learn the protocol to conduct screening, consent, and baseline data collection. Half of them attended a second training day to learn the protocol for intervention delivery. Follow-up technical assistance included mock calls and quality assurance reviews provided by Emory and North Carolina university-based staff to ensure implementation fidelity. I&R specialists conducted study activities in addition to their regular 2-1-1 duties under a sub-contract from the university intended to offset the cost of specialists’ time spent on project activities.

After providing standard 2-1-1 service and screening out callers in crisis, the trained I&R specialists introduced the study to their callers, completed eligibility screening and enrolled those who provided oral consent. Eligible participants were age 18 or older, able to speak and understand English, a smoker living with at least one non-smoker (including children) or a nonsmoker living with at least one smoker, and living in a home that did not have a total smoking ban. One person per household was enrolled in the study.

### Ethics Statement

The study protocol, including verbal consent procedures, was approved by the Institutional Review Board of The University of North Carolina-Chapel Hill (#13–1808). Oral, rather than written consent was obtained because all study recruitment occurred when potential participants called the 2-1-1 I&R telephone line for assistance with other issues. Oral consent and the date, time, and name of recruiting I&R specialist were documented using the study’s online data collection and tracking application. This trial is registered with ClinicalTrials.gov number NCT01868672.

### Data Collection Procedures

All aspects of the study, including eligibility screening, interviews, intervention delivery and follow-up were managed via the study’s custom-built online data collection application, designed to guide staff through all tasks from recruitment through enrollment, program delivery, and follow-up evaluation. Immediately after enrollment and while the caller was still on the line, a 2-1-1 I&R specialist administered a brief baseline interview. After completion of the baseline interview, the data collection application randomly assigned participants to control or experimental conditions.

Trained 2-1-1 I&R specialists delivered the intervention to intervention group participants, and university-based staff blinded to participants’ assigned condition completed follow-up telephone interviews, lasting approximately 20–30 minutes, at 3 and 6 months post-baseline. Participants received a $25 incentive for each interview completed including baseline, 3 month, and 6 month interviews.

When conducting coaching calls and follow-up interviews, a protocol was followed to reach participants including up to 12 call attempts, 2 mailed letters, and up to 6 attempts to reach an emergency contact. Interviews were recorded and reviewed for quality control purposes to ensure adherence to study protocols and implementation fidelity, including all calls until the interviewer completed five consecutive satisfactory interviews, and 10% of calls thereafter.

### Intervention Description

Designed for both smokers and non-smokers, the intervention consisted of three mailings and a 15–20 minute coaching call delivered over a six-week period, in 2-week intervals following the baseline interview: mailing 1, coaching call, mailing 2, and mailing 3. Intervention materials, based on the theme “Some Things Are Better Outside”, focused on creating and implementing a smoke-free home policy following a five-step process.[5, 8] The intervention was rooted in social cognitive theory, stages of change of the transtheoretical model including aspects of persuasion, role modeling, goal setting, environmental cues, and written and verbal reinforcement of actions taken to create a smoke-free home.[45, 46] Messages emphasized smoke-free homes (i.e. smoking outside) rather than smoking cessation. A detailed description of the intervention has been published.[5, 8] The content of the intervention mailings and coaching call were the same as those used for the efficacy trial in Atlanta with very minor modifications, including branding for North Carolina.[5]

### Measures

The measures used in this trial were based on previously validated standardized survey items described in detail in the Atlanta trial’s outcomes paper.[5]

Primary outcome measure: The primary outcome measure was the self-reported presence of a full home smoking ban, assessed at all three time points. Respondents had a full ban if smoking was not allowed anywhere inside their home, a partial ban if smoking was allowed in some places or at some times, and no ban that smoking was allowed anywhere or there were no rules in the home.[47]

Secondary outcome measures: Secondary outcomes, not overtly targeted by the intervention but measured because they were possible spillover effects, included self-report of participants’ SHS exposure in the home, smoking bans in vehicles, and how often someone in the household talked about making the home smoke-free.[30, 48] Among smokers, cessation attempts, number of cigarettes smoked per day, and self-efficacy for quitting also were measured.[38, 39, 49]

Descriptive measures. At baseline, smoking status and demographic characteristics were collected, including participant’s gender, race/ethnicity, employment status, household income, education level, marital status, age, and household composition. Additionally, at 3- and 6- month follow-up participants were asked about enforcement issues; “How often are your smoking rules broken by someone?” with response options never, rarely, sometimes, and very often.

Intervention process measures. During the 3-month interview, intervention group participants who reported having received the coaching call and/or mailings were asked the proportion of materials reviewed (not any, some, most, all), their relevance and usefulness (not at all, a little, somewhat, very), the relevance and usefulness of and satisfaction with the coaching call (not at all, a little, somewhat, very), as well as whether they had taken intermediate behavioral actions recommended by the intervention (e.g., listing reasons to have a smoke-free home, posting the pledge).

Passive air nicotine monitors, utilized in the previous Atlanta trial after the 3-month interview, validated self-reported home smoking bans, but were also found to be challenging and costly to implement and were, therefore, omitted from this trial.

### Power and Statistical Analyses

Statistical power analyses were conducted with SAS 9.2 (SAS Institute, Cary, NC) using the GEE-Size macro. Minimum sample size was estimated assuming a .10 difference between intervention and comparison groups in the proportion of homes that adopt a smoking ban. Rho and psi parameters were included to adjust for autocorrelation between adjacent observations; the dampened exponential correlation structure was used for the correlation structure among repeated measures, and two-sided alpha = .05.

A total sample size of 340 (with 170 participants each in treatment and control groups) would have achieved 80% statistical power to detect a difference of 10% between control and intervention groups to implement a family smoking ban over the 12-month study period. Ultimately we recruited a conservatively large sample of 500 participants.

The analysis plan replicated that of the Atlanta trial. Descriptive statistics of all variables of interest were calculated. Univariate and bivariate distributions were examined for all relevant variables for each time point. Hierarchical Linear Growth Modeling (HLGM) was used to assess the intervention impact on all primary and secondary outcomes (intent-to-treat) using binary logistic (full/no full ban), ordinal logistic (full/partial/no ban), Poisson (days exposed to SHS, # of quit attempts), and linear (number of cigarettes per day, confidence to quit smoking, talked about SFH) multilevel models.[50] HLGM allows modeling of all available data (intent-to-treat) and thus includes all participants for whom at least baseline data are available, i.e. all randomized participants. The analyses modeled linear change over time (which was appropriate based on preliminary investigation of trajectories), as well as a cross-level interaction effect of time and group assignment to model the effectiveness of the intervention. Two sensitivity analyses making more conservative assumptions were employed. The first sensitivity analysis assumed that all participants who did not have follow-up data failed to make their home smoke-free. The second sensitivity analysis also assumed that those who reported enforcement problems did not make their home smoke-free. The same growth curve models were used for the sensitivity analyses. In addition, we investigated patterns of missing data as well as group differences due to randomization for all baseline demographics. Analyses were conducted using SAS 9.4, and HLM7.

## Results

### Participants

**Fig 1:**
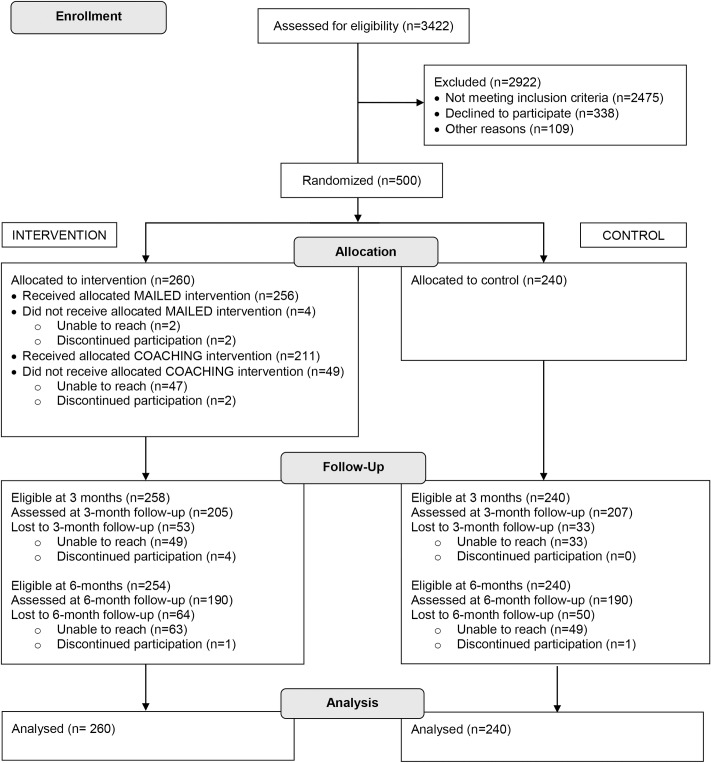
Consolidated Standards of Reporting Trials flow diagram for randomized effectiveness trial: minimal intervention to promote smoke-free homes among 2-1-1 callers, North Carolina, 2013.

Of 3,422 callers assessed for eligibility, 27.7% were eligible, of whom 15.5% declined to participate or ended the call before completing eligibility screening or the enrollment process. The remaining 84.5% (n = 500) were randomized into control or intervention conditions (Fig 1).

Participants were mostly female (87.2%) and African American (61.4%) (Table 1). The majority were smokers (76.6%). Half of homes had no smoking restrictions and half had a partial ban. The majority (79.8%) lived with at least one child younger than age 18 in the home. Intervention and control groups were similar on all baseline variables measured, except the intervention group had more households where all adults were smokers (45.8%) than the control group (35.8%).

Follow-up was completed at 3 months by 82.4% (n = 412) and at 6 months by 76.0% (n = 380) of enrolled participants (Fig 1). Participants lost to follow-up did not differ from those who completed follow-up with one exception: there were more African American participants (79.7%) retained for the 6-month follow-up than Whites (70.2%), *p* = .02 (McNemar test).

**Table 1:** Demographic Characteristics of Study Participants at Baseline: Brief Intervention to Promote Smoke-Free Homes Among 2-1-1 Callers, North Carolina, 2013.

Characteristic	Total (n = 500),	Intervention (n = 260),	Control (n = 240),
	No. (%) or	No. (%) or	No. (%) or
	Mean ±SD	Mean ±SD	Mean ±SD
Gender			
Male	64 (12.8)	34 (13.1)	30 (12.5)
Female	436 (87.2)	226 (86.9)	210 (87.5)
Race/Ethnicity			
African American/Black	301 (61.4)	150 (58.8)	151 (64.3)
White	151 (30.8)	83 (32.6)	68 (28.9)
Other	38 (7.8)	22 (8.6)	16 (6.8)
Employment			
Employed	162 (32.4)	90 (34.6)	72 (30.0)
Unemployed	154 (30.8)	70 (26.9)	84 (35.0)
Homemaker/retired/disabled/other	184 (36.8)	100 (28.5)	84 (35.0)
Income			
≤ $10,000	232 (48.4)	110 (44.2)	122 (53.0)
$10,001–$20,000	89 (18.6)	52 (20.9)	37 (16.1)
$20,001–$35,000	80 (16.7)	50 (20.1)	33 (14.3)
$35,001–$50,000	17 (3.6)	11 (4.4)	6 (2.6)
≥ $50,001	11 (2.3)	2 (0.8)	9 (3.9)
Education			
Less than/some high school	99 (19.8)	52 (20.0)	47 (19.6)
High school graduate/GED	181 (36.2)	83 (31.9)	98 (40.8)
Vocational/technical school/some college	180 (35.0)	104 (40.0)	76 (31.7)
College graduate or higher	40 (8.0)	21 (8.1)	19 (7.9)
Marital status			
Not married, living with partner	143 (28.6)	80 (30.8)	63 (26.3)
Married	105 (21.0)	52 (20.0)	53 (22.1)
Single	252 (50.4)	128 (49.2)	124 (51.7)
Age, y	39.7 ±11.65	39.9 ±11.28	39.6 ±12.06
Smoking status			
Nonsmoker	117 (23.5)	65 (25.1)	52 (21.7)
Smoker	382 (76.6)	194 (74.9)	188 (78.3)
Number of cigarettes per day^1^	13.7 ±8.3	13.9 ±8.49	13.6 ±8.13
Number of smokers in the home			
1	240 (48.0)	131 (50.4)	109 (45.4)
2	180 (36.0)	86 (33.1)	94 (39.2)
≥3	80 (16.0)	43 (16.5)	37 (15.4)
Number of nonsmoking adults in the home			
0	205 (41.0)	119 (45.8)	86 (35.8)
1	207 (41.4)	98 (37.7)	109 (45.4)
≥2	88 (17.6)	43 (16.5)	45 (18.8)
Children in the home			
Children < 18 y in the home	399 (79.8)	210 (80.8)	189 (78.8)
Children < 5 y in the home	179 (35.8)	95 (36.5)	84 (35.0)
Children < 1 y in the home	28 (5.6)	16 (6.2)	12 (5.0)
Home smoking ban status			
Partial ban	252 (50.4)	132 (50.8)	120 (50.0)
No ban	248 (49.6)	128 (49.23)	120 (50.0)

SD = standard deviation. Percentages might not add up to 100% due to rounding or refusal to answer.

^1^For 381 participants who were smokers at baseline.

### Reactions to the Intervention

Of the 260 intervention participants, 98.5% received all three mailings and 81.2% received the coaching call. The four participants not receiving all mailings either voluntarily withdrew from the study or became unreachable. To evaluate reaction to the intervention, we asked follow-up questions during the 3-month interview of intervention participants who reported having received the materials and/or coaching call. In their answers regarding print materials (n = 198) and coaching call (n = 148), 87.4% said intervention mailings were “somewhat” (25.8%) or “very relevant” (61.6%); 92.4% said the materials were “somewhat” (19.2%) or “very useful/helpful” (73.2%). The coaching call was rated as somewhat (32.4%) or very (58.1%) relevant by 90.5%, and somewhat (21.0%) or very (70.3%) useful/helpful and 91.3% of participants respectively; 93.9% said they were “somewhat” (15.5%) or “very satisfied” (78.4%) with their call.

High proportions of intervention participants at 3-month follow-up (n = 198) reported taking intermediate behavioral actions recommended by the intervention: 83.3% reported having a smoke-free homes talk with family, 67.7% used the provided stickers, 61.6% came up with a list of reasons to have a smoke-free home, and 59.6% put up signs. While the intervention did not attempt to get people to quit smoking, 17.2% of participants reported themselves or someone in their household calling smoking cessation services.

### Intervention Impact

Primary outcome; household smoking bans. Participants in the intervention group were more likely to have a smoke-free home at both follow-up points: At 3 months, 38.1% in the intervention group (versus 19.3% in the control group, *p* < .001) had full bans; full ban rates were higher for both groups at 6 months compared to 3 months with significantly more full bans in the intervention group than the control group (43.2% vs. 33.2%, *p* = .02). The longitudinal intent-to-treat analysis showed a significant intervention effect over time (OR = 1.31 per follow-up wave, *p* = .001), i.e. OR = 1.72 at 6 months (Table 2). The sensitivity analyses showed that when defining those lost to follow-up as failures, i.e. not having a full ban, results were still significant at 3 months with 30.0% of intervention participants versus 16.7% of control participants creating a smoke-free home (*p* = .001). However at 6 months, the difference was not statistically significant (31.5% vs. 26.3%, *p* = .19). When defining success even more stringently by restricting success to only those reporting a full ban and no enforcement issues, a similar pattern emerged with significantly more intervention than control group participants with a successful ban at 3 months (15.0% vs. 7.5%, *p* = .01) and non-significant differences between groups at 6 months (20.0% vs. 13.8%, *p* = .06).

**Table 2:** Impact of the Intervention on Primary and Secondary Outcomes at 3 and 6 Months Post Baseline: Intervention to Promote Smoke-Free Homes Among 2-1-1 Callers, North Carolina, 2013.

	3 month assessment			6 month assessment			ITT Analysis Intervention group change^1^	
	Intervention	Control	*p-value*	Intervention	Control	*p-value*	Effect	*p-value*
	(n = 205),	(n = 207),		(n = 190),	(n = 190),			
	No. (%) or	No. (%) or		No. (%) or	No. (%) or			
	Mean ±SD	Mean ±SD		Mean ±SD	Mean ±SD			
**Primary outcome**								
Home smoking ban								
Full ban	78 (38.1)	40 (19.3)	< .001	82 (43.2)	63 (33.2)	.02	1.31	.001
No full ban	127 (62.0)	167 (80.7)		108 (56.8)	127 (66.8)			
Partial ban	76 (37.1)	101 (48.8)	< .0001	74 (39.0)	72 (37.9)	.04	1.34	.002
No ban	51 (24.9)	66 (31.9)		34 (17.9)	55 (29.0)			
**Secondary outcomes—all participants**								
No. of days exposed to SHS in past 7 days	2.5 ±2.96	3.4 ±3.10	.002	2.1 ±2.87	2.9 ±3.12	.006	-0.35	.0004
Car smoking ban^2^			.33			.053	1.22	.03
Full ban	61 (29.8)	49 (23.7)		72 (37.9)	47 (25.3)			
Partial ban	64 (31.2)	65 (31.4)		40 (21.1)	52 (27.4)			
No ban	40 (19.5)	49 (23.7)		39 (20.5)	39 (20.5)			
No car	40 (19.5)	44 (21.2)		39 (20.5)	51 (26.8)			
Talked about SFH^3^	3.0 ±1.05	2.7 ±1.12	.01	3.0 ±1.03	2.8 ±1.16	.04	N/A^4^	N/A^4^
**Secondary outcomes—smokers only**								
Smokers	(n = 139)	(n = 165)		(n = 119)	(n = 142)			
Quit attempts last 3 months	1.4 ±2.1	0.8 ±1.2	.01	2.1 ±1.7	1.8 ±1.1	.61	1.19	.09
No. of cigarettes per day	10.8 ±7.8	12.1 ±7.7	.16	9.7 ±7.3	10.9 ±7.5	.19	-0.62	.03
Confidence to quit smoking	6.7 ±2.6	6.0 ±2.8	.03	6.7 ±2.7	6.3 ±2.8	.22	0.24	.02

^1^ITT analyses are growth models that include data from all participants including those who were not reached for 3 months and/or 6 months follow-up. Effect sizes and p-values reported are those from the cross-level interaction effect between time and group assignment. Effect for full and partial ban is an odds ratio, for exposure to SHS and quit attempts is event rate ratio, and beta for the other outcomes.

^2^No car excluded from analysis.

^3^On a scale from 1 = never to 4 = very often.

^4^ N/A because not assessed at baseline.

Secondary outcomes. At both 3 and 6 months, intervention participants had significantly less self-reported SHS exposure compared to those in the control group (*p* = .001 at 3 months and *p* = .01 at 6 months). Intervention group participants were more likely than control group participants to have a car smoking ban (37.9% vs. 25.3%, *p* = .03).

There were no statistically significant differences in changes in smoking status over time between the two groups, but smokers in the intervention group reported a significantly greater reduction in the number of cigarettes smoked per day than smokers in the control group. Intervention group participants reported significantly more quit attempts and more confidence about quitting at 3 months than control group participants; these differences were not maintained at 6 months.

## Discussion

The Atlanta efficacy trial of the Smoke-Free Homes intervention demonstrated the intervention worked, with more intervention participants having a full home smoking ban at 6 months (40.0%) compared to the control group (25.4%).[5] The current effectiveness trial results were comparable to Atlanta’s outcomes, although with slightly lower reach for the coaching call, indicating that delivery of the program by 2-1-1 I&R specialists, including dose delivered and implementation fidelity, was comparably effective to delivery by university personnel. With training, technical assistance and quality control provided by Emory and North Carolina university-based staff and the support of the Smoke-Free Homes custom-built online data collection and tracking application, 2-1-1 I&R specialists engaged in the subsequent North Carolina trial proved capable of, and enthusiastic about, recruiting for and delivering this intervention.

Demographic characteristics of study participants were consistent with the overall demographics of NC 2-1-1 callers. Given the demographic and somewhat different cultures and tobacco control climates between Atlanta and North Carolina (i.e., North Carolina has a comprehensive smoke-free air law), the two trials conducted to date suggest generalizability across settings and extension of intervention effects when delivered by 2-1-1 staff vs. university personnel. Future research will compare the effects of intervention moderators and intervention population/catchment area differences on intervention results across trials in Atlanta, North Carolina, and a third trial in Texas to further establish generalizability.

In both trials, intervention participants progressed, with increases in proportions of homes moving from no ban to partial ban or from partial ban to full ban by 6-month follow-up. Three months post-baseline, with 83.3% of NC intervention participants reporting they had a talk with their family about adopting a smoke-free home policy and more than half engaging in several other intermediate behavioral actions recommended by the intervention, these are indications that even in cases where participants did not successfully adopt a total home smoking ban, the intervention brought about discussion of a ban and steps toward making changes to home smoking policies and behaviors. These process data also support the intervention’s theory of change with “have a family talk” the second step in the change process. Of note, outcomes were achieved with a less intensive intervention than previously tested interventions involving multiple or more intensive counseling sessions targeting families with young or medically compromised children.[10–15, 42, 43, 51, 52]

While secondary outcomes such as SHS exposure, smoking cessation behavior, and car smoking bans were not direct targets of the intervention, they nevertheless were impacted by the intervention. At both follow-up points, intervention participants had significantly less self-reported SHS exposure than those in the control group. As compared to the control group, intervention participants who smoke also reported more quit attempts, greater confidence to quit smoking, and smoking fewer cigarettes per day. Full or partial car smoking bans, while not addressed directly by the intervention, could be viewed as an extension of a home smoking ban, were reported by significantly more intervention participants at 6 months, and may be considered for inclusion in future interventions.

### Additional findings

An unexpected finding of this study was an observed reactivity effect: to a greater extent in the NC trial than in the Atlanta trial, the proportion of homes in the control group with full bans rose substantially from 3-month to 6-month follow-up interview, from 19.3% to 33.2%. Furthermore, intervention group participants reported significantly more quit attempts than in the control group at 3 months, but no significant difference was found at 6 months. We suspect this may have been an effect of the 3-month interview serving as an intervention itself.

During the 3-month interview, all study participants were asked a series of detailed questions about their home environment and barriers to adopting and enforcing a smoke-free policy that may have prompted them to think about the importance of making their home smoke-free. The interview itself, even in the absence of mailings and coaching call, appeared to encourage families to discuss and adopt smoke-free home policies, supporting the notion that engaging the 2-1-1 client population, whether with a minimal intervention or an interview, can have a substantial impact on their/their family’s health. Our findings suggest that secular changes in the culture supporting tobacco control and reactivity to participation, especially measures, may promote change even without the formal intervention. We have seen similar results in previous trials addressing reduction in SHS exposure, [11–13] but further analyses of pooled trial data are warranted to further explore these findings. They are further supported by Hovell *et al*.’s findings[53] that asthma intervention participants randomized into minimal measures, full measures, and full measures plus treatment for reduction in SHS exposure experienced as large an effect from measures as from treatment.

While differences between intervention and control group’s ban status were diminished by the aforementioned measurement effect, the statistically significant difference between intervention and control groups at 6 months is a good indicator of the program’s effectiveness with intervention participants having 72% higher odds of having a full smoking ban in their home than control participants. These results were achieved despite the intervention group having more households where all adults were smokers (which would likely make achieving a home smoking ban more difficult) than the control group.

This trial was limited by the use of self-report measures without validation of air nicotine monitors, and follow-up assessments may have been inflated by socially desirable responses. In the Atlanta trial, air nicotine monitors confirmed accuracy of self-reports, but were difficult and costly to implement, and therefore were not used in this trial. This deletion, however, moved this trial more fully into the class of a systematic replication and extension toward an effectiveness trial. Furthermore, our final follow-up assessment was conducted 6 months post-baseline, which may be insufficient for assessing long-term effects. We saw, however, increases in bans rather than decreases between our 3- and 6-month assessments, indicating a minimal diminished effect over time.

Overall, this minimal intervention to promote smoke-free home policies was well received by intervention participants and by the 2-1-1 call centers that delivered it. This trial’s findings indicate that 2-1-1 staff could not only recruit participants but deliver the intervention successfully. Of the various types of replication studies identified by Valentine et al. (2011)[19] (i.e., statistical, generalizability, implementation and theory development), this study can be described as an implementation replication study with variation centered on who delivers the intervention. It also includes elements of a generalizability replication with a slightly more diverse study population and a different tobacco control context than in the original efficacy trial. The results of a second effectiveness trial in Texas will provide additional findings regarding the generalizability of the intervention (allowing for pooled data analyses across trials) setting the stage for a national dissemination program. This Smoke-Free Homes research program and the related series of replication studies serves as a model for translational research following efficacy trials conducted by traditional university researchers.

## Supporting Information

S1 CONSORT Checklist (DOC)

S1 Trial Protocol Brief Intervention to Create Smoke-Free Home Policies in Low-Income Households: North Carolina Effectiveness Trial. (PDF)
