# Supplementary material for: A Minimal Intervention to Promote Smoke-Free Homes among 2-1-1 Callers: North Carolina Randomized Effectiveness Trial
Source: PLoS One. 2016 Nov 2;11(11):e0165086. doi: 10.1371/journal.pone.0165086 (PMC5091897; doi:10.1371/journal.pone.0165086)
Supplement: S1 Trial Protocol — (PDF) [file pone.0165086.s002.pdf]

Smoke-Free Homes Project

Brief Intervention to Create Smoke-Free Home Policies in Low-Income Households: North Carolina Effectiveness Trial

PI: Rebecca Williams, MHS, PhD
Co-I: Kurt Ribisil, PhD

1.0 Purpose
The purpose of this study is to test an intervention designed to create smoke-free homes in low income households. The main hypothesis to be tested is that a higher proportion of households in the intervention group will establish and maintain a smoke-free home than in the measures-only control group.

1.1 Background
Due in large part to the remarkable progress in creating smoke-free environments in the US over the past two decades, the home is now a primary source of exposure to secondhand smoke for both children and nonsmoking adults.[1] The prevalence of smoke-free homes has increased rapidly in recent years. From 1992 to 2008, the proportion of smoke-free homes increased from 43.2% to 78.1%.[2, 3] Rules that limit smoking in the home are less common in households in which at least one person smokes and in African American and low income households.[4-6] As a result, exposure to secondhand smoke remains relatively high for African Americans, children, and low-income families across racial and ethnic groups.[7] Intervention research that identifies effective and practical solutions in reaching low-income families has considerable potential to reduce disparities related to tobacco use. Assisting low-income households to go smoke-free will reduce exposure to secondhand smoke, help smokers to quit and potentially disrupt the smoking initiation process in adolescents.[8-10]

<u>Health Effects of Secondhand Smoke Exposure</u>: Secondhand smoke (SHS) is a known carcinogen with substantial evidence linking it to lung cancer.[1, 11-13] Approximately 3,400 lung cancer deaths are attributable to SHS annually in the U.S.[14] Exposure to SHS increases risk of lung cancer by about 20% in nonsmokers who live with a smoker, with risk increasing with the years of exposure to a smoker and the number of cigarettes smoked.[1, 15, 16] Evidence linking SHS to other cancers is limited, although several studies have shown associations between SHS exposure and breast cancer.[1, 11, 14] SHS exposure also causes coronary heart disease, accounting for between 30,000 and 70,000 deaths in the U.S. each year.[1, 17, 18] Reviews of the evidence linking SHS to coronary heart disease consistently show increased risks of 27-30% among nonsmokers who live with a smoker.[1, 19] In addition, evidence that SHS may cause strokes is beginning to accumulate.[11, 20] Exposure to SHS can also exacerbate asthma and underlying lung disease, contribute to respiratory problems, and reduce lung function in adults.[11, 21] Chronic exposure to secondhand smoke in children is related to increased risk of lower respiratory infections (e.g., bronchitis and pneumonia), middle ear infections, severity of asthma symptoms, sudden infant death syndrome, and lung cancer later in life.[1, 12, 14, 22-24] Risk for adverse health effects in children increases as the number of adult smokers in the household increases.[25]

Smoke-Free Homes and Exposure to SHS in the Home: Despite significant declines in exposure to SHS over the past decade due to restrictions on smoking at work and other public places, children and nonsmoking adults who live with a person who smokes still experience significant exposure to SHS in the home.[7, 25] Based on an analysis of NHANES data from 1999-2004; Schober and colleagues reported that 10.2% of the US nonsmoking population self-reported home SHS exposure, with 46.4% showing detectable serum cotinine levels, a biomarker for nicotine exposure.[7] Detectable serum cotinine levels were most common in children 4-11 years of age (60.5%) and adolescents (55.4%). Among adults 20 years of age and above, 42.2% were exposed to SHS. Notably, significant disparities in SHS exposure exist by race; 70.5% of African American nonsmokers have detectable serum cotinine, compared to 43% of whites and 40.0% of Mexican Americans.[7]

Smoke-free homes have been shown to reduce exposure to SHS for both nonsmokers and children.[23, 26-28] Wakefield and colleagues found that total smoking bans in the home were associated with lower cotinine levels among children with asthma and at least one parent who smoked, relative to children living in homes with less restrictive home smoking environments.[26] In addition, partial bans provided more protection from SHS than unrestricted smoking in the home. Similarly, other studies have shown that smoking restrictions in the home were associated with less SHS exposure among both adolescents and adults.[27, 28]

Additional Benefits of Smoke-Free Homes: In the past decade, evidence has accumulated to show that household smoking restrictions have the same effect in aiding cessation as do worksite smoking bans.[29, 30] Smokers who live in homes with either a partial or total ban have been shown to be more likely to attempt to quit smoking and to successfully quit, with total bans having a stronger effect than partial bans. [4, 8, 31-34] A recent review of smoke-free homes and adult smoking behavior concluded, "There is strong and consistent population-level evidence that a smoke-free home is associated with increased smoking cessation and decreased cigarette consumption in adult smokers" (p. 1131).[35] Mills and colleagues also suggested that smoking cessation interventions consider the inclusion of smoke-free homes as a cessation strategy.[35]

Further, household smoking bans are an important component of anti-smoking socialization. In a study of the relationship of anti-smoking socialization and smoking onset in children, Jackson and Henriksen found that household smoking bans were associated with delayed smoking onset.[36] Because the age at which a child first tries smoking predicts smoking later in life, interventions that can delay smoking onset may ultimately contribute to lower prevalence of smoking.[37] Constant exposure to secondhand smoke may also increase a child's tolerance for smoke, resulting in a less negative physical reaction to their first experimentation with cigarettes. Wakefield and colleagues found that home smoking bans were associated with reduced transition through the smoking uptake process in teens, meaning that teens living in homes with smoking bans were less likely to become regular smokers than teens in homes with no bans.[10] Clark et al. also reported reduced odds of adolescent smoking in households with strict smoking bans.[9]

1.2 Preliminary Studies

Interventions to Reduce SHS Exposure in the Home: Two Healthy People 2020 objectives address SHS exposure; one calls for reducing the proportion of children who are regularly exposed to tobacco smoke at home, and the other calls for a reduction in the proportion of

nonsmokers who are exposed to SHS.[38] To date, intervention research to reduce SHS in the home has largely been focused on reducing the SHS exposure of children, often children with asthma, using cessation and/or indoor smoking control as the protection strategy.[23, 39] Interventions have been set in clinics or homes. A 2003 review of 19 studies of interventions that attempted to reduce SHS among children concluded that although more rigorous study designs were needed, intervention effects had been demonstrated (average effect size=0.34, Cohen's d). This review found that clinic-based interventions were most common (k=10) and were typically brief interventions with a recommendation to reduce SHS exposure together with printed educational materials. Home-based interventions (k=8) were typically more intensive, usually involved 5-7 30-minute sessions over several months, and were more likely to be theoretically grounded. These reviewers concluded that more intensive interventions and interventions with an explicit theoretical framework (Social Cognitive Theory, behavioral modification, operant condition theory, and motivational interviewing)[39-43] demonstrated greater effectiveness.

More recent studies have shown that more intensive interventions are not consistently successful in reducing SHS exposure.[44, 45] Given the need for greater "reach" for SHS interventions if they are to produce a population effect, Hovell & Hughes' recently placed special importance on brief interventions, despite their likely smaller effect sizes.[46] Applied on a large scale, brief interventions, even with modest effect sizes, have more potential to achieve population-level impact than intensive interventions. Brief interventions that focus in detail on how to achieve and maintain household smoking rules are rare, even when the goal is reduced SHS exposure in the home. The proposed intervention focuses directly on creating a smoke-free home, with a referral to the state Quitline as the primary cessation-oriented message. In conclusion, brief interventions with explicit instructions for establishing smoke-free homes have not been adequately tested and are a high priority for future research due to their potential for broad scale dissemination.

<u>Qualitative Research on the Process of Creating a Smoke-Free Home</u>: From 2003-2006, Drs. Kegler and Escoffery (Co-Investigator) conducted a qualitative study of the <u>decision-making process</u> families go through in adopting a smoke-free home. The research, Tobacco Use in Rural Families (TURF) involved in-person interviews with adults in 90 rural African American and White households in Southwest Georgia with a child aged 10-14. Briefly, it was found that across all types of households, family discussions about smoking bans focused heavily on protecting children.[47-49] In homes with one or more adult nonsmokers, the smell and dangers of secondhand smoke were also highlighted. Households with both a smoker and a nonsmoker discussed the nonsmoker's aversion to breathing smoke. Women and nonsmokers were most likely to initiate family discussions about creating a smoke-free home. Additional triggers for initiating a ban included: changing household composition (i.e., a new baby, marriage/divorce), health problems and related physician recommendations, moving to a new home, and a family member quitting smoking. Disagreements about smoking rules took the form of verbal resistance, active resistance or negotiated compromise, but were relatively rare.

<u>Preliminary results of the intervention pilot study:</u> A pilot study was conducted to test the feasibility, acceptability and short-term outcomes of a brief, four-component intervention for promoting smoke-free home policies among low-income households. Forty participants (20 smokers and 20 non-smokers) were recruited to receive the intervention at two week intervals. Half of the participants were randomly selected to place an air nicotine monitoring device in their

homes. The design was a pre-test post-test with follow-up at two weeks post intervention. The primary outcome measure was self-reported presence of a total home smoking ban. At follow-up, 77.8% of participants reported having tried to establish a smoke-free rule in their home, with significantly more non-smokers attempting a smoke-free home than smokers (*p* = .03). These attempts led to increased smoking restrictions, i.e. going from no ban to a partial or total ban, or from a partial to a total ban, in 42.5% of the homes. At follow-up, 33.3% of the participants reported having made their home totally smoke-free. Additionally, smokers reported smoking fewer cigarettes per day. This study served to refine a proposed intervention that will affect a larger population in the present full-scale trial. The results and experience gained in the pilot study helped in the design and implementation process of the present intervention for smoke-free homes among low income populations. Results suggest that the intervention is promising and warrants a rigorous efficacy trial.

1.4 Significance

This research is significant because 1) it addresses a setting in which tobacco policy and intervention research is less developed at the population level, 2) it will contribute to interventions to create smoke-free homes for underserved populations who have higher smoking rates and less presence of total bans, and 3) it will also address cancer and chronic disease health disparities. It also builds on Drs. Escoffery's and Kegler's formative research on the decision-making process and messages in establishing a smoke-free home and our pilot study to test the intervention messages and educational materials. Better intervention trials to create total home smoking bans are needed. Such interventions have to be simple, cost effective and not too time-consuming, so that they will be adopted on a large scale and have a major impact on public health.

2.0 Design

2.1 Sample

We will recruit 500 participants from North Carolina to participate in a randomized controlled trial. The following criteria must be met for participation: 1) only one participant per household will be recruited; 2) be 18 years of age or older; 3) speak and understand English; 4) either be a smoker with at least one other non-smoking person living in the household OR a non-smoker who lives with a smoker; and 5) not currently have a total smoking ban.

2.2 Setting

All study procedures, including informed consent and data collection, will be done over the telephone from the offices of United Way of North Carolina 2-1-1, University of North Carolina Center for Health Promotion and Disease Prevention, and the Rollins School of Public Health, Emory University.

2.3 Recruitment

United Way of North Carolina 2-1-1 (NC 2-1-1) will be responsible for recruiting study participants for the trial and recruitment will occur on a rolling basis. CITI certified line agents will assist in the recruitment of study participants. These line agents' primary role is to answer incoming calls to NC 2-1-1 and provide information and referrals that connect people in need with important community resources such as food and utility assistance, housing and health

care. The line agents will be answering incoming calls at random and screening every caller that meets the eligibility criteria for screening (i.e. callers may not be in immediate crisis, such as facing homelessness or domestic violence cases).

2.4 Description of Intervention

The smoke-free homes intervention consists of four components, three mailings of print materials and one coaching call, aimed at increasing household smoking bans and reducing secondhand smoke exposure. The materials are designed to target both smokers and nonsmokers who allow smoking in the home. The conceptual model is based on social cognitive theory [50, 51] and the transtheoretical model's stages of change. [52-54] Social cognitive theory was selected because of its emphasis on both cognitive and environmental determinants of behavior, and the interplay between them known as reciprocal determinism [55]. The intervention targets proximal determinants of behavioral capacity, self-efficacy, and outcome expectations related to creating a smoke-free home, and smoking behaviors. Although not well-studied with respect to smoke-free homes, these variables have been shown as important in a wide range of behavioral interventions based on social cognitive theory. [55] Through the use of persuasion, role modeling, goal setting, environmental cues and reinforcement, change strategies tied to social cognitive theory, participants are encouraged to work through the five steps of creating a smoke-free home. These include: 1) deciding to create a smoke-free home, 2) talking to household members about making a home smoke-free, 3) setting a date for going smoke-free, 4) actually making a home smoke-free, and 5) keeping the home smoke-free. Because the five-steps aligned quite well with stages of change as articulated in the Transtheoretical Model, stages of change was also included in the conceptual model.[54] This allows us to focus the coaching component of the intervention on the appropriate step (or stage) for each participant.

The five steps emerged from prior qualitative work on creating smoke-free homes (e.g., factors influencing the decision to go smoke-free, the need to talk to household members about a possible rule, challenges in enforcing the rule), combined with existing smoke-free home campaigns by the U.S. Environmental Protection Agency and Health Canada.[56, 57] This formative research helped develop intervention messages, for example, on common reasons to create a smoke-free home. Participant ideas for promoting a smoke-free home, which included environmental strategies such as posting no smoking signs in the home, helping the smoker find a comfortable place outside to smoke, and removing ashtrays and lighters, were also included in the educational materials. Finally, we asked about barriers to enforcing a ban. These barriers, such as feeling uncomfortable or concern over showing disrespect to a visitor or older relative were acknowledged in the materials as well, along with potential solutions.

All print materials were designed around the theme of "Some Things are Better Outside." The first component, mailed after completion of the baseline survey, is a "tool-kit" for creating a smoke-free home. The tool-kit includes a "*Five-Step Guide to a Smoke-Free Home"* which describes the steps, tips, and strategies in planning for, making, and keeping a smoke-free home. The guide is packaged in a 9"x 12" mailer that folds out to 18"x 24" when opened. The mailer is designed to be interactive and educational to include definitions of secondhand smoke and smoke-free homes; a list of reasons to have a smoke free home; truths about secondhand smoke; a tear off pledge participants and household members could sign after deciding to make their home smoke-free; and two tear off Smoke-Free Home signs with adhesive tape strips.

The second component of the intervention is a Five Step Coaching Call. The coaching script incorporates the five steps as described in the "*Five-Step Guide to a Smoke-Free Home.*" The semi-structured script elicits responses on the progress of making the home smoke-free, benefits of a smoke-free home and challenges and barriers to setting a smoke-free home rule. A stage of change assessment is performed (i.e. have no interest in making home smoke-free, are thinking about making home smoke-free, decided to make home smoke-free, or already have a smoke-free home) to prompt the counselor to provide stage-based messages. The coaching session ends with a summary of the call and goals for making and/or keeping a smoke-free home.

The third component includes additional educational information in the form of a photo story which depicts a household comprising a mother, grandmother and a child going through the process of making their home smoke-free. It provides information on secondhand smoke and its dangers, tips on having a conversation with the smoker in the home, finding ways of making smoking outside easier and celebrating being smoke-free. Also included in this mailing is a "*Challenges and Solutions: Keeping your Home Smoke-Free"* booklet. It provides ten most commonly reported challenges derived from our formative research (e.g. you are not the head of the household and you can't make the rules in your home; you live in an apartment and there is no porch or yard to use as a smoking area, etc.) and offers easy to implement solutions.

The fourth component includes a newsletter with testimonials and success stories portraying families and their reasons to have a smoke-free home as well as examples of ways to keep their home smoke-free. This mailing also includes a thirdhand smoke fact sheet and six smoke-free home stickers that could be used as reminders to smoke outside (i.e. placed on bathroom mirrors, cigarette packs, ashtrays, etc).

2.5 Procedures & Study Design

A randomized-controlled trial with two-group repeated measures design will be conducted. Data will be collected at baseline, three-months, and six-months post baseline to test the intervention to create smoke-free homes in low-income households. There will only be one person recruited per household that meets our eligibility criteria and that same participant will participate in the collection of the baseline and follow-up surveys. Participants will be randomized into one of two groups, either intervention or control. Those in the intervention group will also receive all components of the intervention as described above. Control group participants will participate in data collection activities at baseline, 3, and 6-month follow-up.

As mentioned earlier, participants will initially call 2-1-1 to receive referral services and all participants who are not in immediate crisis will be invited to participate in the study. Line agents will provide a brief description of the project and all interested participants will be screened for eligibility. All eligible participants will be verbally consented to participate. Informed consent will include a description and purpose of the study, procedures, risks and benefits, freedom to withdraw, compensation, confidentiality, and contact information for study staff and the UNC-Chapel Hill IRB. All those who verbally consent to participate in the trial will complete the baseline survey by telephone and be enrolled in the study. We expect this initial call to last 15 to 20 minutes and participants will receive a $25 gift card for completing the baseline interview.

Callers interested in the project that are not able to remain on the phone to complete the consenting process and request a callback to do so, will be given a return call by a line agent for

the opportunity to complete the consent process. Name and phone number of the caller will be collected by the line agent for callback purposes only. Callers will be enrolled in the study only after IRB approved informed consent has been completed.

Randomization
Immediately after the completion of the baseline interview, participants will be randomized into intervention and control conditions. Investigators and data collection staff will be blind to group assignment until data analysis is complete. Randomization will be as follows:

Randomization of eligible and consented participants into 2 groups	
Treatment Group (intervention) (n=250)	Control Group (control) (n=250)

Intervention Delivery
Following randomization, intervention participants will receive Component #1 – Mailing #1 (as described earlier) by mail. Then, two weeks later, intervention research staff will deliver Component #2 – Five-Step Coaching Call. At week 4, intervention delivery staff will mail Component #3 – Mailing #2 and at week 6, participants will receive Component #4 – Mailing #3.

Follow-up Data Collection
Blinded research interviewers will call participants to complete the 3-month (week 12) and 6-month (week 24) follow-up interview. The measures are described below. Interviews are expected to last approximately twenty to thirty minutes and participants will receive a $25 gift card for completing the baseline and follow-up interview, respectively.

Study activities & timeline

**Timeline**	**Data collection point/ Steps**
Week 0	• Initial 2-1-1 call, screening, consent, and baseline data collection<br>• Randomize enrolled participants into 2 groups
Immediately after randomization	• Intervention Delivery; Mailing #1 (treatment group only)<br>• Week 2 – Coaching Call (14 days after mailing #1)<br>• Week 4 – Mailing #2 (14 days after coaching call)<br>• Week 6 – Mailing #3 (14 days after Mailing #2)
Week 12 – (12 weeks from baseline interview)	• 3 month follow-up
Week 24 – (24 weeks from baseline interview)	• 6 month follow-up

Audio-recordings
A small sample of baseline interviews, coaching calls, and follow-up interviews will be audio-recorded for quality control purposes (approximately 10% of each type). Audio recording files

will be stored electronically on the secure and password protected Research drive. Once the study and data analysis is complete, audio files will be deleted.

<u>Web-based Study Management Application (Smoke-Free Homes Tracking Tool)</u>
A secure web-based enrollment, data collection, and project management application will be used. The Smoke-Free Homes Tracking Tool will aid in tracking study participants from screening through intervention delivery and data collection. This Tracking Tool will be used by line agents, intervention delivery staff, research interviewers, project management staff, and data analysts each with its own level of applicable access. All staff related to this research study and who will access the Tracking Tool will be CITI certified and IRB approved.

The Tracking Tool will be used first by 2-1-1 line agents to screen callers for participation in the study. All calls taken by each agent will be tracked by the Tracking Tool, including their reason for calling, whether they were actively screened for inclusion in the study or not. This will allow us to analyze callers' reasons for calling and for refusing to participate (where applicable), allowing for better understanding of the recruitment process.

The Tracking Tool will prompt the line agent to administer a brief screening survey (uploaded to IRB application) to determine eligibility for the study. If the caller is eligible for participation, the application will provide a consent form to be read to the caller. If the caller agrees to participate, the application will provide a baseline survey to be administered by the agent, then randomize the participant into the intervention or control group. The Tracking Tool will prompt study staff to mail intervention materials at predetermined intervals as described in the intervention delivery section above.

The application will also aid in intervention delivery. Each day, the system will prompt line agents delivering the intervention with a list of study participants who need materials. The system will also indicate which materials intervention staff should send. The application will keep track of participants who have been mailed materials. Likewise, the application will prompt intervention staff to call study participants at the appropriate interval to offer the Five-Step Coaching session, and will guide the staff through the session and allow them to record information and collect data about the interaction. This system will allow for the tracking and management of all aspects of the intervention recruitment and delivery in a single secure database. The Tracking Tool will provide researchers with multiple ways of processing and accessing this data: they will be able to review all records for individual participants and quickly determine at which point in the intervention/data collection process participants are. It will provide reports on progress towards recruitment and other intervention goals. Data will be stored in an Access database, which can easily be exported for analysis to Excel, SAS, or SPSS. See section 2.8 for details on data analysis.

2.6 Measures
The primary outcome measure for this study is the self-reported presence of a total home smoking ban. The baseline interview will include questions related to secondhand smoke exposure, smoking history, cigarette consumption, cessation attempts, beliefs about secondhand smoke, stage of change and self-efficacy for quitting, and car smoking restrictions. Demographic information will also be collected at baseline. This includes respondent's ethnicity, age, gender, education level, marital status, household income, employment status, and household composition. Additionally, the proportion of family members and friends who smoke, and the number of smokers and non-smokers in the home will be assessed.

The 3- and 6-month follow-up interviews will be similar to the baseline interview. Additional questions will include measures related to secondhand smoke reduction behaviors, beliefs regarding secondhand and thirdhand smoke, ban enforcement, and stage of change for smoke-free home ban. The 3-month interview will also include process measures to assess the receipt of mailed materials, the proportion of materials read, the usefulness and relevance of the materials, and satisfaction with the Five-Step Coaching Call.

The screening and eligibility questionnaire, baseline and follow-up interviews, and informed consent form are uploaded for IRB review and approval.

2.7 Risks and Benefits to participation

We will not be discussing any sensitive topics and we do not expect any of the telephone calls, survey questions, or print materials to cause any harm or distress. Participants may benefit from having a smoke-free home as a result of our intervention materials.

2.8 Data Analysis

SPSS and the SAS statistical package for PC will be used for data management and the analyses. The primary analyses of the data will be performed according to the participants' original intervention assignment (intent-to-treat principle). All data from all participants randomized will be included in the final analysis. Baseline demographic characteristics of participants will be summarized overall and by intervention group stratified by smoking status. Baseline characteristics will be compared between the control and intervention groups as well as smokers and non-smokers.

The main outcome for this RCT is the adoption of a smoking ban in the home. Change in smoking ban status due to the intervention will also be examined. We will also perform sub-group analyses to see if the intervention effect is different for specific sub-populations. Sub-populations of interest include smokers versus non-smokers and households with children versus those with no children. Secondary outcomes will also be examined. We will examine differences between intervention groups on the air nicotine levels in the home. For smokers, we will look at differences of assignment to the intervention on cessation attempts and number of cigarettes smoked. Validation of self-report with air nicotine monitors will be included. Furthermore, process evaluation data will be examined descriptively and will be selected in analysis on intervention effectiveness.

3.0 Training

All personnel involved in data collection and intervention delivery will be trained prior to beginning the study. All 2-1-1 study personnel involved in recruitment and informed consent will receive explicit training on the relevant processes. Similarly, 2-1-1 study personnel involved in intervention delivery (intervention delivery staff) will be trained on the specific strategies used to facilitate the call and elicit interactions with call recipients; how to effectively communicate with and motivate participants; how to assess and address any barriers that may be in the way of creating a smoke-free home. All data collection trainings will be conducted by research staff members, from both UNC-Chapel Hill and Emory University, who assisted in the development of the survey questions and have extensive experience with data collection by telephone and the informed consent process. All study team members have completed the necessary CITI certifications to maintain human subject protection.

4.0 Plans for data management and monitoring

All study staff, including the PI, will participate in the monitoring of participant data. The proposed protocol contains minimal risk so no adverse events are expected. However, should any adverse event occur, the study staff member who discovered the adverse event would immediately alert the Principal Investigator, who will have the responsibility of informing the UNC IRB. The PI will meet regularly with study staff to ensure that data security is maintained and data analysis is handled as outlined above. Each study participant will be assigned an ID number and surveys will be identifiable via assigned IDs only. A separate Excel database will link identification codes with participants' names.

5.0 Confidentiality

Confidentiality of participant data will be protected at all times. The following procedures will take place:

a) All study personnel will be thoroughly trained on how to maintain confidentiality.
b) Each participant will be assigned a unique ID number. We will use each participant's ID number instead of his/her name whenever we can, including the surveys and during data analysis. Once the data is exported from the Tracking Tool, we will ensure that all personally identifying information, such as names, will not be stored in the same electronic data file as other study data.
c) Contact information obtained from participants will be kept on a secure and HIPAA compliant web-based database. No hard copies of data will be stored.
d) All data collected will be backed-up on a secure "Research" drive and will only be accessible to CITI certified study staff.
e) Participant names and other facts that might identify a specific participant will not appear when we present this study or publish its results.
f) A sample of baseline interviews, Five-Step Coaching calls, and follow-up interviews will be audio-recorded for quality control purposes. We will use digital recorders and files will be stored electronically on the secure and password protected "Research" drive. Once the study procedures and data analysis is complete, audio files will be deleted.

6.0 Informed consent

We will use an IRB approved informed consent prior to conducting any study procedures. Since most of the study procedures are telephone-based and we would not have any physical contact with the participants, the informed consent process will also occur by telephone. Documentation of informed consent will occur in the Tracking Tool and personnel involved with the process will document the date and time of consent, and provide an electronic signature.

During the informed consent process, we will describe the purpose of the study, procedures, risks and benefits to participating, compensation, and how confidentiality will be maintained. Since contact information for study PI and Project Manager will be provided, a hard copy of the consent form will be mailed to all participants who request it. Member of the research team will be available to explain any questions regarding the study and content of the informed consent form.

7.0 Plans to inform participants of new findings or research results

Our current plans for informing study participants of new findings include posting study related information and results on the UNC Center for Health Promotion and Disease Prevention website. This will be done once study procedures seize and all data has been collected and analyzed.

8.0 References

1. US Department of Health and Human Services, *The health consequences of involuntary exposure to tobacco smoke: A report of the surgeon general.*, 2006, US Department of Health and Human Services, Centers for Disease Control and Prevention, Coordinating Center for Health Promotion, National Center for Chronic Disease Prevention and Health Promotion, Office on Smoking and Health.: Atlanta, GA.
2. Centers for Disease Control and Prevention, *State-Specific prevalence of smoke-free home rules-United States, 1992-2003.* Morbidity and Mortality Weekly Report, 2007. **56**(20): p. 501-504.
3. Centers for Disease Control and Prevention, *State-specific secondhand smoke exposure and current cigarette smoking among adults - United States, 2008.* Morbidity and Mortality Weekly Report, 2009. **58**(44): p. 1232-1235.
4. Gilpin, E.A., et al., *Home smoking restrictions: which smokers have them and how they are associated with smoking behavior.* Nicotine & Tobacco Research, 1999. **1**(2): p. 153-62.
5. Norman, G.J., et al., *Smoking bans in the home and car: Do those who really need them have them?* Preventive Medicine, 1999. **29**(6 Pt 1): p. 581-9.
6. Koepke, D., B.R. Flay, and C. Johnson, *Health behaviors in minority families: The case of cigarette smoking.* Family & Community Health, 1990. **13**(1): p. 35-43.
7. Schober, S., et al., *Disparities in secondhand smoke exposure.* MMWR Morb Mortal Wkly Rep, 2008. **57**(27): p. 744-747.
8. Messer, K., et al., *The effect of smoke-free homes on smoking behavior in the U.S.* American Journal of Preventive Medicine, 2008. **35**(3): p. 210-6.
9. Clark, P.I., et al., *Impact of home smoking rules on smoking patterns among adolescents and young adults.* Preventing Chronic Disease, 2006. **3**(2): p. A41.
10. Wakefield, M.A., et al., *Effect of restrictions on smoking at home, at school, and in public places on teenage smoking: cross sectional study.[see comment][erratum appears in BMJ 2000 Sep 9;31(7261):623].* BMJ, 2000. **321**(7257): p. 333-7.
11. International Agency for Research on Cancer (IARC), *Evaluating the effectiveness of smoke-free policies*, in *Handbooks of Cancer Prevention, Tobacco Control*, W.H. Organization, Editor 2009: Lyon, France.
12. United States Environmental Protection Agency, Office of Health Environmental Assessment, and Office of Research and Development, *Respiratory Health Effects of Passive Smoking: Lung Cancer and Other Disorders*, 1992.
13. Janerich, D.T., et al., *Lung cancer and exposure to tobacco smoke in the household.[see comment].* New England Journal of Medicine, 1990. **323**(10): p. 632-6.
14. California Environmental Protection Agency: Air Resources Board. *Proposed identification of environmental tobacco smoke as a toxic air contaminant*. 2005 [cited 2009 12/27/2009]; Available from: http://escholarship.org/uc/item/8hk6960q.
15. Taylor, R., F. Najafi, and A. Dobson, *Meta-analysis of studies of passive smoking and lung cancer: effects of study type and continent.* Int J Epidemiol, 2007. **36**(5): p. 1048-59.
16. Hackshaw, A.K., M.R. Law, and N.J. Wald, *The accumulated evidence on lung cancer and environmental tobacco smoke.[see comment].* BMJ, 1997. **315**(7114): p. 980-8.

17. Institute on Medicine Committee on Secondhand Smoke Exposure and Acute Coronary Events, *Secondhand Smoke Exposure and Cardiovascular Effects: Making Sense of the Evidence*, 2009, National Academies Press: Washington, DC.
18. Pitsavos, C., et al., *Association between exposure to environmental tobacco smoke and the development of acute coronary syndromes: the CARDIO2000 case-control study.* Tobacco Control, 2002. **11**(3): p. 220-5.
19. Law, M.R., J.K. Morris, and N.J. Wald, *Environmental tobacco smoke exposure and ischaemic heart disease: an evaluation of the evidence.[see comment].* BMJ, 1997. **315**(7114): p. 973-80.
20. Zhang, X., et al., *Association of passive smoking by husbands with prevalence of stroke among Chinese women nonsmokers.* Am J Epidemiol, 2005. **161**(3): p. 213-8.
21. Eisner, M.D., et al., *Environmental tobacco smoke and adult asthma. The impact of changing exposure status on health outcomes.[retraction in Eisner MD, Shiboski SC, Yelin EH, Blanc PD, Bainton DF. Am J Respir Crit Care Med. 2000 Sep;162(3 Pt 1):788; PMID: 10988082].* American Journal of Respiratory & Critical Care Medicine, 1998. **158**(1): p. 170-5.
22. McMartin, K.I., et al., *Lung tissue concentrations of nicotine in sudden infant death syndrome (SIDS).* Journal of Pediatrics, 2002. **140**(2): p. 205-9.
23. Gehrman, C.A. and M.F. Hovell, *Protecting children from environmental tobacco smoke (ETS) exposure: a critical review.* Nicotine & Tobacco Research, 2003. **5**(3): p. 289-301.
24. Anderson, H.R. and D.G. Cook, *Passive smoking and sudden infant death syndrome: Review of the epidemiological evidence.[erratum appears in Thorax 1999 Apr;54(4):365-6].* Thorax, 1997. **52**(11): p. 1003-9.
25. Pirkle, J.L., et al., *Trends in the exposure of nonsmokers in the U.S. population to secondhand smoke: 1988-2002.* Environmental Health Perspectives, 2006. **114**(6): p. 853-8.
26. Wakefield, M., et al., *Restrictions on smoking at home and urinary cotinine levels among children with asthma.* American Journal of Preventive Medicine, 2000. **19**(3): p. 188-92.
27. Pizacani, B.A., et al., *Household smoking bans: which households have them and do they work?* Preventive Medicine, 2003. **36**(1): p. 99-107.
28. Biener, L., et al., *Household smoking restrictions and adolescents' exposure to environmental tobacco smoke.* Preventive Medicine, 1997. **26**(3): p. 358-63.
29. Jeffery, R.W., et al., *Restrictive smoking policies in the workplace: effects on smoking prevalence and cigarette consumption.* Preventive Medicine, 1994. **23**(1): p. 78-82.
30. Brenner, H. and A. Mielck, *Smoking prohibition in the workplace and smoking cessation in the Federal Republic of Germany.* Preventive Medicine, 1992. **21**(2): p. 252-61.
31. Farkas, A.J., et al., *The effects of household and workplace smoking restrictions on quitting behaviours.* Tobacco Control, 1999. **8**(3): p. 261-5.
32. Okah, F.A., *Effect of children on home smoking restriction by inner-city smokers.* Pediatrics., 2002. **109**(2): p. 244-249.
33. Kegler, M.C. and L.H. Malcoe, *Smoking restrictions in the home and car among rural Native American and white families with young children.* Preventive Medicine, 2002. **35**(4): p. 334-42.
34. Pizacani, B.A., et al., *A prospective study of household smoking bans and subsequent cessation related behaviour: the role of stage of change.* Tobacco Control, 2004. **13**(1): p. 23-8.
35. Mills, A.L., et al., *The effect of smoke-free homes on adult smoking behavior: a review.* Nicotine Tob Res, 2009. **11**(10): p. 1131-41.
36. Jackson, C. and L. Henriksen, *Do as I say: Parent smoking, antismoking socialization, and smoking onset among children.* Addictive Behaviors, 1997. **22**(1): p. 107-114.

37. Chassin, L., et al., *The natural history of cigarette smoking: predicting young-adult smoking outcomes from adolescent smoking patterns.* Health Psychology, 1990. **9**(6): p. 701-16.
38. US Department of Health and Human Services. *Healthy People 2020*. 2011; Available from: http://www.healthypeople.gov/2020/default.aspx
39. Hovell, M.F., et al., *Reduction of environmental tobacco smoke exposure among asthmatic children: a controlled trial.[erratum appears in Chest 1995 May;107(5):1480].* Chest, 1994. **106**(2): p. 440-6.
40. Greenberg, R.A., et al., *Evaluation of a home-based intervention program to reduce infant passive smoking and lower respiratory illness.* Journal of Behavioral Medicine, 1994. **17**(3): p. 273-90.
41. Wahlgren, D.R., et al., *Reduction of environmental tobacco smoke exposure in asthmatic children: a 2-year follow-up.* Chest, 1997. **111**(1): p. 81-88.
42. Hovell, M.F., et al., *Effect of counselling mothers on their children's exposure to environmental tobacco smoke: randomised controlled trial.[see comment].* BMJ, 2000. **321**(7257): p. 337-42.
43. Emmons, K.M., et al., *A randomized trial to reduce passive smoke exposure in low-income households with young children.* Pediatrics, 2001. **108**(1): p. 18-24.
44. Conway, T.L., et al., *Intervention to reduce environmental tobacco smoke exposure in Latino children: null effects on hair biomarkers and parent reports.* Tob Control, 2004. **13**(1): p. 90-2.
45. Zakarian, J.M., et al., *Behavioral counseling for reducing children's ETS exposure: implementation in community clinics.* Nicotine & Tobacco Research, 2004. **6**(6): p. 1061-74.
46. Hovell, M.F., et al., *Counseling to reduce children's secondhand smoke exposure and help parents quit smoking: a controlled trial.* Nicotine Tob Res, 2009. **11**(12): p. 1383-94.
47. Kegler, M.C., et al., *A qualitative study of how families decide to adopt household smoking restrictions.* Family & Community Health, 2007. **30**(4): p. 328-41.
48. Kegler, M.C., C. Escoffery, and S. Butler, *A qualitative study on establishing and enforcing smoking rules in family cars.* Nicotine & Tobacco Research, 2008. **10**(3): p. 493-7.
49. Escoffery, C., M.C. Kegler, and S. Butler, *Formative research on creating smoke-free homes in rural communities.* Health Education Research, 2009. **24**(1): p. 76-86.
50. Bandura, A., *Social Foundations of Thought and Action: A Social Cognitive Theory*1986, New York: Prentice-Hall.
51. Bandura, A., *Social Learning Theory*1977, Englewood Cliffs, NJ: Prentice-Hall.
52. Prochaska, J.O., et al., *Measuring processes of change: applications to the cessation of smoking.* Journal of Consulting & Clinical Psychology, 1988. **56**(4): p. 520-8.
53. Prochaska, J.O., C.C. DiClemente, and J.C. Norcross, *In search of how people change. Applications to addictive behaviors.* American Psychologist, 1992. **47**(9): p. 1102-14.
54. Prochaska, J., Redding, C, and Evers, K, *The transtheoretical model and stages of change.*, in *Health Behavior and Health Education, 4th edition*, B.R. K. Glanz, K. Viswinath, Editor 2008, Jossey-Bass, Inc.: San Francisco.
55. McAlister, A., Perry, C., and Parcel, G., *How individuals, environments, and health behaviors interact.* , in *Health Behavior and Health Education, 4th edition*, B.R. K. Glanz, K. Viswinath, Editor 2008, Jossey-Bass, Inc.: San Francisco.
56. US Environmental Protection Agency, *Smoke-Free Homes and Cars Program*.
57. Health Canada. *Make your home and car smoke-free: A guide to protecting your family from second-hand smoke.*; Available from: http://www.hc-sc.gc.ca/hc-ps/pubs/tobac-tabac/second-guide/index-eng.php.
